# Health Care Equity in the Use of Advanced Analytics and Artificial Intelligence Technologies in Primary Care

**DOI:** 10.1007/s11606-021-06846-x

**Published:** 2021-05-23

**Authors:** Cheryl R. Clark, Consuelo Hopkins Wilkins, Jorge A. Rodriguez, Anita M. Preininger, Joyce Harris, Spencer DesAutels, Hema Karunakaram, Kyu Rhee, David W. Bates, Irene Dankwa-Mullan

**Affiliations:** 1 Brigham and Women’s Hospital, Boston, USA; 2 Vanderbilt University Medical Center, Nashville, USA; 3 IBM Watson Health, Cambridge, USA

## Abstract

The integration of advanced analytics and artificial intelligence (AI) technologies into the practice of medicine holds much promise. Yet, the opportunity to leverage these tools carries with it an equal responsibility to ensure that principles of equity are incorporated into their implementation and use. Without such efforts, tools will potentially reflect the myriad of ways in which data, algorithmic, and analytic biases can be produced, with the potential to widen inequities by race, ethnicity, gender, and other sociodemographic factors implicated in disparate health outcomes. We propose a set of strategic assertions to examine before, during, and after adoption of these technologies in order to facilitate healthcare equity across all patient population groups. The purpose is to enable generalists to promote engagement with technology companies and co-create, promote, or support innovation and insights that can potentially inform decision-making and health care equity.

Primary care has a critical role to play in ensuring that mission-driven values aimed at eliminating health care disparities are prioritized in the development, selection, clinical implementation, and use of advanced analytics and AI technologies. Because the application of these technologies in primary care is in its infancy, primary care professionals have a unique opportunity to guide the growth of fair, transparent, and ethical AI and analytics applications that embody health equity principles that meet the needs of diverse populations.

Today, clinical decision-making in primary care is influenced by the ongoing integration of advanced analytics and AI technologies into the practice of medicine.^1^ Examples include patient risk stratification, predictive modeling for disease progression,^2,3^ decision-support applications,^4,5^ and population health management tools for cancer screenings,^6,7^ diabetes,^8,9^ cardiovascular disease,^10–12^ and other chronic disease conditions.^13^ These and other similar tools may or may not explicitly address the needs of diverse patient populations in primary care. Unless explicit strategies are used to promote equity, advanced analytics may inadvertently perpetuate inequities in primary care delivery, such as the use of algorithms that erroneously treat race categories as biological rather than social attributes in clinical decision making.^14^

The importance of articulating equity as a specific goal for integrating AI into care is described in the 2019 National Academy of Medicine (NAM) report, *Artificial Intelligence in Health Care: The Hope, The Hype, The Promise, The Peril.* The report describes a quintuple aim to improve population health, reduce costs, improve the patient experience, promote care team well-being and achieve health care equity.^15^ Specifically, the report suggests that embracing health care equity would challenge a siloed approach to health care by addressing the diversity of patient needs using varied sources of data that include social determinants of health and psychosocial risk factors (Fig. 1). Equity, integral to the quintuple aim, would also require engaging diverse stakeholders to inform the design of AI applications and to monitor the impact of these technologies. The NAM report underscores the need for explicit strategies to actively embrace health care equity; without such strategies, AI applications are likely to reflect human biases in ways that will widen inequities by race/ethnicity, gender identity, sexual orientation, disability status, age, social class, geography, and other dimensions of social identity.^15,16^

**Figure 1:**
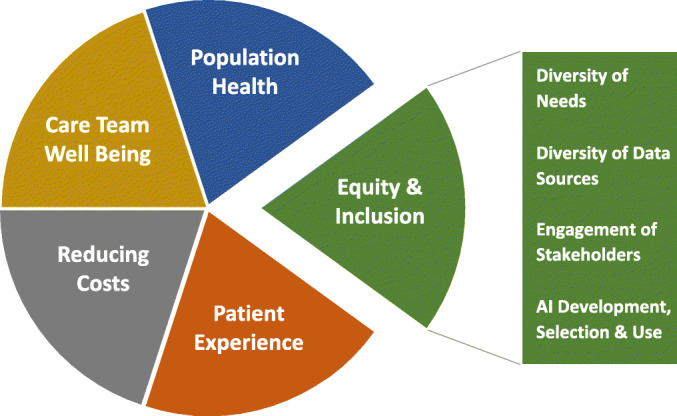
Building on the quintuple aims of equity and inclusion in health and healthcare (National Academy of Medicine).^14^

Indifference to technology and passive acceptance of biased tools pose risks to health care equity among diverse groups. To prevent this, we must be willing to articulate the priorities for successful AI and advanced analytics implementation and adopt strategies and processes that lead to equitable outcomes. To further these aims, we propose the following series of questions that should be considered before and during the adoption of an AI technology or advanced analytic strategy into practice. First, what needs of diverse patient populations can be better served by applying advanced analytics and AI technology? How can novel and diverse data sources be leveraged to enhance equity in AI implementations? How can patients and community members engage with stakeholders involved in shaping the use of AI in the delivery of health care? And finally, how are principles of diversity and inclusion reflected among those who are involved in the development, selection, and use of technology solutions to enable equitable health care?

## UNDERSTANDING THE NEEDS OF DIVERSE POPULATIONS

**Figure 2:**
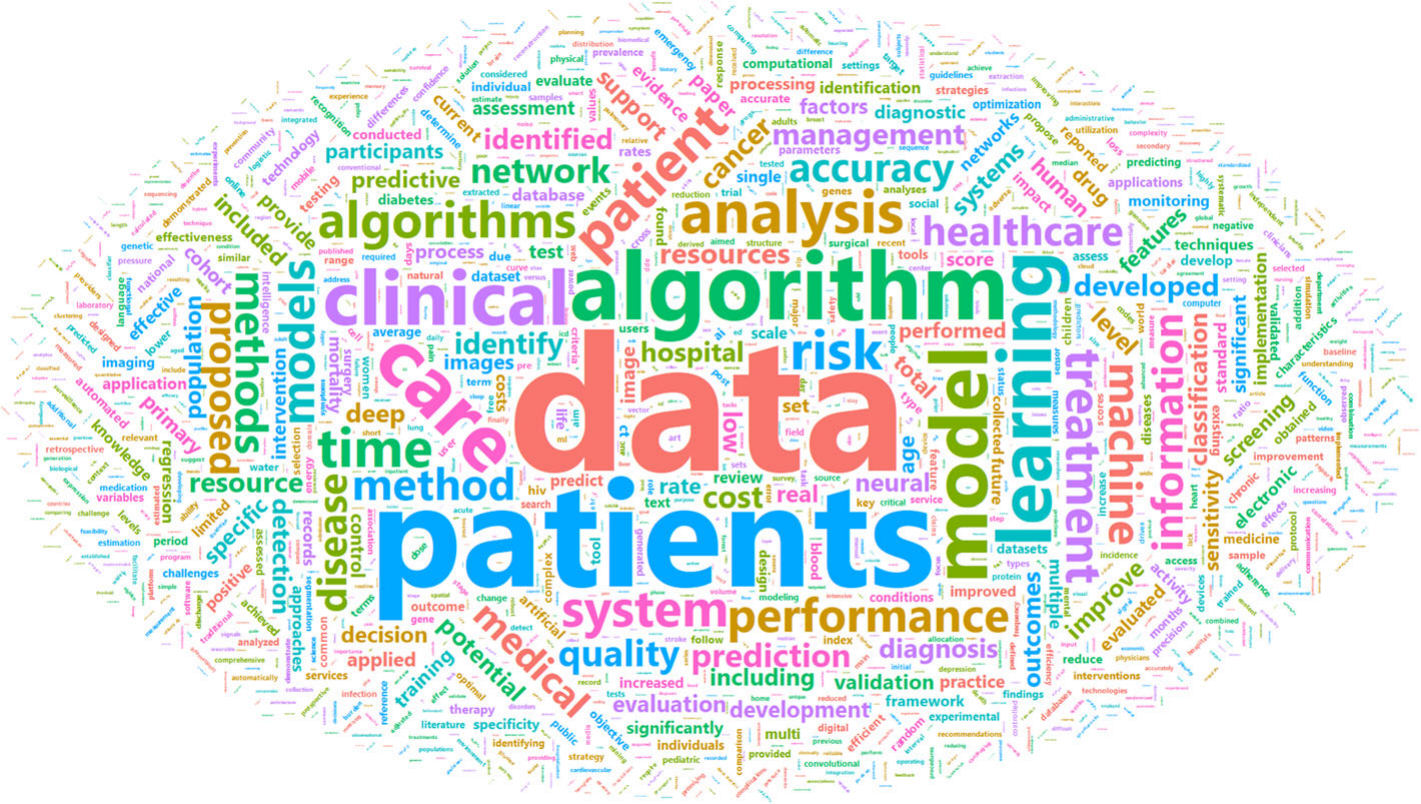
Visualizing the opportunity to focus on diversity and health equity. The figure shows the authors’ own qualitative analysis of a PubMed search for articles published in 2019. The text analysis displays the frequency of words used in the title and abstracts of 2994 published articles in 2019 that use AI tools and methods related to health care delivery. Words related to diversity, disparities, equity, and related terms were found infrequently.

The ideal implementation of advanced analytics and AI technologies should democratize health care and actively address problems that are relevant to the health needs of socially marginalized populations who face structural racism and discrimination in access to care, resulting in disparate health outcomes.^15^ Position papers and special academic journal issues, as well as white papers from government, academic, and commercial bodies, are raising awareness of current longstanding issues related to health care equity.^17,18^ Yet, we note that the existing academic medical literature employing AI-based techniques has rarely focused on diversity, health equity or health care disparities (Fig. 2).

When used to explicitly address health care equity, AI techniques can help identify and prevent disease progression in diverse populations. For example, deep learning models have been used to improve detection of diabetic retinopathy in multiethnic populations.^19^ As we seek more equitable technology solutions to improve health care delivery, we must actively seek to understand and offer solutions relevant to the needs of all patients, especially patients in historically underserved or marginalized groups.

## DIVERSE DATA SOURCES FOR EQUITABLE AI

To fully embrace equity as a part of health care delivery, we must understand the patient experience within and outside of clinical encounters. Data sources used in primary care decision making must not only reflect clinical data but also incorporate social determinants of health (SDOH)—where patients are born, grow, work, live, and age. Within primary care settings, advanced analytic tools or predictive models may draw data from electronic health records (EHR), claims, and clinical trial data, while data outside the clinical encounter are not captured routinely or in a standardized way. If the social context is not adequately represented in the data that informs tool design, the development of decision-support tools may inappropriately influence primary care delivery, with repercussions for practice management, unfavorable treatment variation among patients with complex needs, or continuity of care following hospitalization admissions.

Although there is a movement to better understand the social context in primary care, particularly in accountable care risk contracts,^20^ other primary care practices may not be obligated to collect or report patient demographics or SDOH data. For example, Klinger et al. reported up to 20% of Spanish language-preferring patients were misidentified as preferring English.^21^ Barriers to collecting SDOH data in primary care include the need to develop workflows to collect data, a lack of experience using patient-centered tools developed to collect SDOH data, and the need for standardized processes and resources to respond to needs identified through data collection.^22^ Barriers to better collection of data that reflect the social experiences of patients will necessarily limit the insights that can be gained from claims or EHR-derived AI analytics and may have broad implications for the development of diagnostic and treatment algorithms that promote health equity. Furthermore, even when data related to equity or social determinants of health are considered, this information can lead to unexpected bias in algorithms if not appropriately integrated and interpreted. As primary care embraces a more data-driven landscape, the structure, purpose, and manner of data generation become increasingly critical questions that deeply influence the ability to use technology to foster equitable care for patients. Incentives for SDOH data collection through Medicaid Accountable Care organizations (ACOs) has made data more available in primary care settings; additional strategies are needed to support these efforts.^23^

In addition to SDOH data and data from the clinical encounter, novel data sources such as mobile or remote sensing devices can provide valuable insights. Incorporating these data would require active patient and community partnerships to ensure equitable access to these devices and inclusion of data into clinical research and practice. For example, SDOH screening efforts at the clinic level in closed-looped data systems can augment geographic data on service location from AI-based tools.^24^ Increasingly, large research cohort studies, such as the National Institutes of Health (NIH) *All of Us* Research Program provides tools for integrating novel, participant-collected data sources from diverse groups as a part of the NIH’s Precision Medicine Initiative.^25^

## PATIENT AND COMMUNITY ENGAGEMENT

Importantly, AI and advanced analytics implementation in primary care must be a collaborative effort that involves patients and communities from diverse social, cultural, and economic backgrounds in an intentional and meaningful manner. Integrating the perspectives of those with lived experience is necessary to frame problems and define success. Convening community advisory boards and patient advisory panels are established methods for engaging patients and community stakeholders. Innovative strategies include community engagement studios,^26^ which are project-specific consultative sessions that allow patients and community members to provide reactions and feedback to specific approaches proposed by health care professionals and scientists. Additional strategies, such as the use of Patient-Reported Outcomes Measures (PROMs), are increasingly used in EHRs to assess healthcare delivery outcomes from the patient perspective.^27,28^ Innovations in the collection of patient-designed PROMS can ensure that data needed to implement AI solutions incorporate metrics of success that are designed by diverse patient groups.

## DEVELOPMENT, SELECTION, IMPLEMENTATION, AND USE OF EQUITABLE TECHNOLOGIES

Those who fund and purchase AI-based technologies and analytics—including primary care clinicians—strongly influence which types of products and algorithms gain widespread use and ultimately impact patients. Funders and purchasers can also serve as gatekeepers for technologies that might worsen existing disparities. With $131 billion in venture capital investments going to healthcare startups in 2018 alone,^29^ primary care leaders have an opportunity to assess and fund new tools based not only on their potential financial performance but also on their impact on populations subject to inequities in health care. Those investing in technology systems, such as EHRs used in primary care, have the responsibility of promoting policies and working with industry partners that reflect and appreciate the value of data science technology that is focused on health equity.^30^

Primary care clinicians, managers, and organizations all participate in the technology ecosystem through critical roles as collaborators in the development and innovation of new tools, users and purchasers of technology, and employers of information technology (IT) teams. Although IT and implementation teams often play important roles influencing product design, application, and ultimately patient experience, their roles are often overlooked in the process of the design, implementation, and use of equitable health care. Leaders involved in selection of technology must not only be cognizant of structural racism and other inequities experienced by their patient populations but also understand and communicate to developers how technology may be used to address these inequities. Team member characteristics such as race, ethnicity, country of origin, immigrant status, gender, age, sexual orientation, geography, education, culture, religion, cognition patterns, and primary language, as well as learning and working styles, are all reflected in the iterative design of software tools,^31^ including tools used in clinical settings. It is crucial to consider whether elements of the aforementioned diversity factors have been integrated into the development and implementation of AI and advanced analytics,^32,33^ which can be facilitated by investing in a diverse, local IT workforce. A diverse team is particularly important to support the design of strategies to monitor and manage bias in the development and application of advanced analytics and AI algorithms. Process architecture and frameworks are needed to define what constitutes fairness and reduction of bias, as well as to inform interventions that eliminate biases before the technology is used. Unless development, implementation, and IT team members represent multiple dimensions of diversity present within a community, it is almost impossible to uncover the necessary questions needed to fairly and equitably represent the needs of the population that the technology is intended to serve.

## THE WAY FORWARD

The responsibility to examine outcomes for diverse patient groups is a joint one that must be undertaken comprehensively by all primary care stakeholders. It is critically important to monitor the use of advanced analytics and AI technology to ensure that benefits are accruing to diverse groups in an equitable manner. The values and metrics of success must be clearly defined, and technologies selected need to be instrumented to collect such values and metrics. By using SDOH as well as novel data sources, and by explicitly monitoring results of advanced technology implementations in diverse populations (Table 1), developed and implemented by a diverse workforce, health care providers can ensure their efforts reflect the equity they hope to achieve in health care and use such measurements in iterative development and continual improvement processes aimed at health equity.

**Table 1:** Strategies That Can Be Used to Achieve Equity in Primary Care AI Implementations

Goal	Strategies
Ensure AI technologies improve health and health care of diverse populations, as defined and articulated by the populations who experience structural racism and structural inequities.	1. Engage patients and communities as key stakeholders in articulating equitable outcome measures
	2. Define health care equity as a key success metric for technology implementation
	3. Provide training for primary care clinicians and administrators in the use of AI for health equity promotion
Incentivize “ecosystems” to produce AI technologies that promote health care equity	4. Select industry partners that prioritize health equity as an outcome of AI tool development and implementation
	5. Create diversity within teams that develop AI solutions
Create systems of accountability for achieving measurable processes and outcomes of equitable care provision	6. Collect data that reflect priorities of diverse populations, including SDOH and PROMs
	7. Monitor process measures and improvement in health outcomes along the lines of race/ethnicity and other aspects of social identity using health equity dashboards

One way to accomplish these goals is by using analytical tools such as dashboards that are configured to compare practice performance across groups, stratified by race, gender, zip code, and other dimensions related to equity. A best practice would require examining the use of advanced analytics and AI tools in rigorous pilots prior to full implementation of any system. At minimum, model performance should be evaluated along social dimensions that are known to be associated with decreased access to equitable care.

By engaging patients and community partners, driving diversity and inclusion in development, implementation and IT teams, purchasing from and forming intentional partnerships with health equity-focused industry partners, and by assessing metrics before, during, and after implementation, primary care professionals can lay the groundwork for selection, implementation, and use of technologies aligned to health care equity for their patient population. To truly foster leadership in these issues, primary care clinicians and administrators may need to expand training and medical education on uses of AI tools for health equity promotion.

## CONCLUSIONS

The opportunity to achieve health equity through advanced analytics and AI tools challenges us to hold ourselves accountable for our role as participants and drivers of technology development. Innovative approaches to development and implementation are needed if we are to realize the promise of reducing health disparities with the help of AI and advanced analytics. To accomplish this vision, we must embrace proactive strategies to intervene and monitor our progress towards achieving the goal of equitable health care in primary care settings. The actions needed to address health care equity in AI and advanced analytics are not technical; instead, they necessitate the collaboration of primary care professionals and the populations that they serve to drive the use of technology in ways that fulfill the promise of health equity.
